# Developmental regression and movement disorder as a phenotypic variant of POLR3A Mutation—Case report

**DOI:** 10.1002/ccr3.6556

**Published:** 2022-11-15

**Authors:** Ali Nikkhah, Sepideh Rezakhani

**Affiliations:** ^1^ Pediatric Neurology Research Center Mofid Children's Hospital, Shahid Beheshti University of Medical Sciences Tehran Iran

**Keywords:** dystonia, leukodystrophy, POLR3A, striatum

## Abstract

POLR3A is a main subunit encoding RNA polymerase III, which is involved in transcription of many RNA structures. Here, we report a new presentation of c.1771‐6C > G intronic variant presenting as developmental regression, seizure, and dystonia in a 6‐year‐old boy associated with striatum involvement in the brain MRI.

## Graphical abstract

A new presentation of POLR3A gene mutation presenting as developmental regression, seizure and dystonia in a 6‐year‐old boy associated with striatum involvement in the brain MRI.

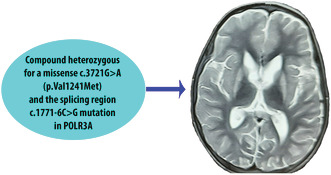

## 1 INTRODUCTION

POLR3A (RNA Polymerase III Subunit A) is a protein‐coding gene, which is responsible for the fundamental transcription of tRNA, mitochondrial RNA‐processing RNA, 5 S ribosomal RNA, H1 RNA, and noncoding RNAs. Considering that this gene is involved in the transcription of many RNA structures, its mutations can lead to a wide range of phenotypes. The two main phenotypic categories with a variety of presentations are hypomyelinating leukodystrophy‐7 (HLD7), and a rare neonatal progeroid syndrome (NPS) also known as Wiedemann‐Rautenstrauch syndrome (WDRTS).^1^, ^2^

HLD7 is an autosomal recessive leukodystrophy mostly presenting as early‐onset hypomyelination, hypogonadotropic hypogonadism, hypodontia, spasticity, dystonia, and neurodevelopmental regression. WDRTS is commonly presented as considerable prenatal and severe postnatal growth retardation, facial dysmorphism, dental anomalies (natal teeth and hypodontia), and generalized lipodystrophy along with abnormal fat distribution. Progressive ataxia and tremor have also been reported as a separate phenotype in some cases.^3^, ^4^

Furthermore; in recent years, variants of POLR3A mutations without predominant ataxia have been reported as well. These variants manifest as striatal disorders mostly presenting with dystonia and involvement of putamen, caudate and red nucleus. Also, biallelic POLR3A variants have been reported as a cause of hereditary spastic ataxia.^5^, ^6^, ^7^, ^8^

In this report, we present a 6‐year‐old boy with a history of developmental regression, seizure, and dystonia from the age of two, who was diagnosed as a phenotypic variant of POLR3A mutation through whole‐exome sequencing.

## 2 CASE PRESENTATION

**FIGURE 1:**
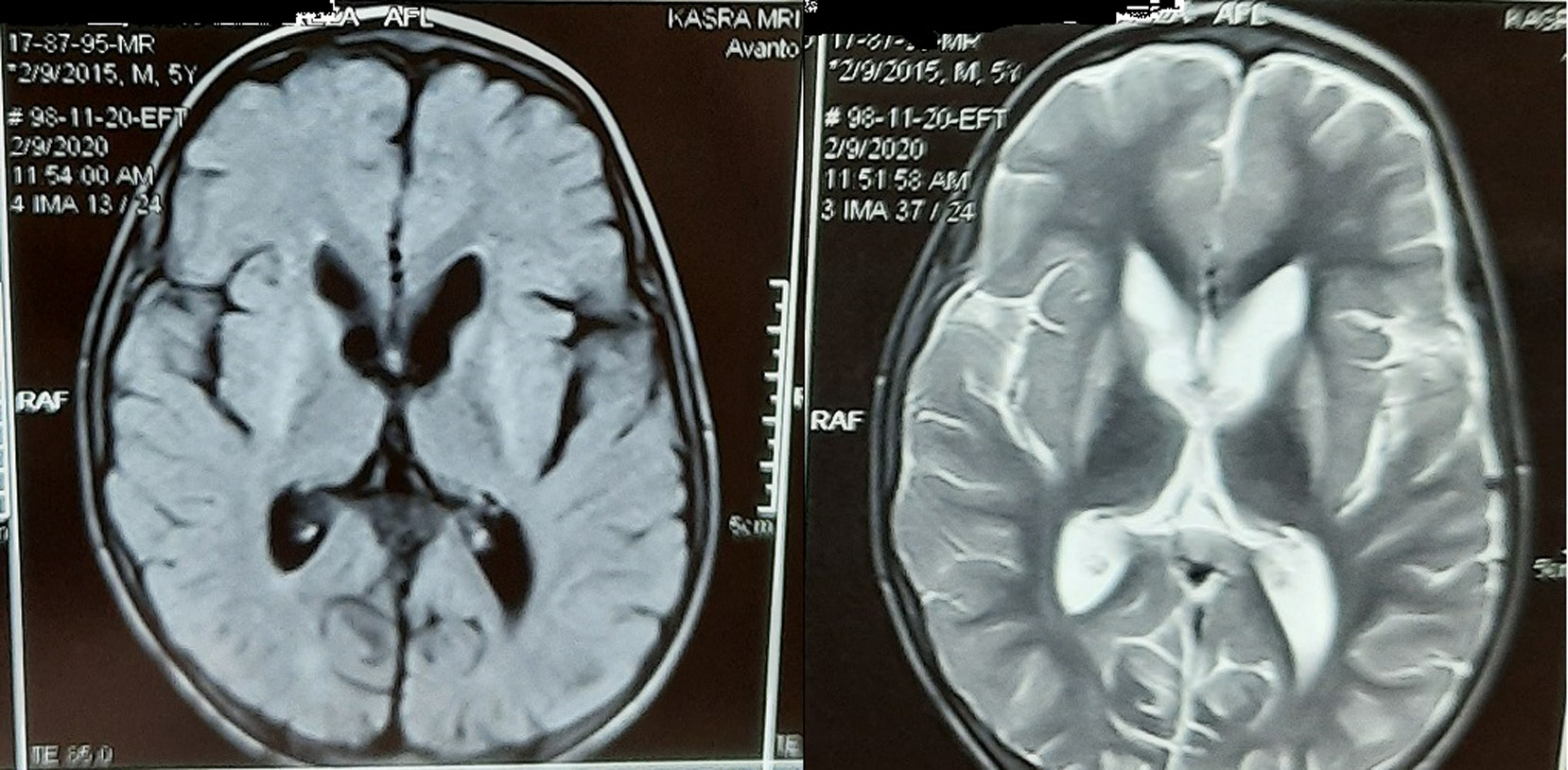
Axial FLAIR MR image (left) and axial T2W image (right) show abnormal high signal changes in bilateral caudate and putamen (striatum)

The patient was a 6‐year‐old boy with an uneventful prenatal and birth history. He was born to non‐consanguineous parents. His siblings are normal. He was first referred to pediatric neurologist with inability to walk at age 18 months. He developed seizure associated with fever at two‐year‐old followed by gradual developmental regression and recurrent unprovoked tonic clonic seizures together with upward gaze lasting for about 1 min. Consequently, he developed lack of ocular fix and follow and head control together with severe swallowing problem, failure‐to‐thrive, mild axial hypotonia, generalized dystonia, severely delayed cognition, and no speech in addition to motor developmental delay. Head circumference, dentation, and ophthalmoscopic examination were within normal limits. The first brain MRI and metabolic studies were within normal limits as well. EMG‐NCV was performed due to decreased deep tendon reflex and hypotonia which was normal. No interictal epileptiform discharges were found in two routine EEGs. With deterioration of his condition, second brain MRI was performed which revealed bilateral striatal involvement (Figure 1).

Finally, whole‐exome sequencing (WES) was performed in January 2019 which was reported as follows: compound heterozygote mutation in POLR3A gene (intron 13: c.1771‐6C > G, exon 31: c.4037G > A p.C1346Y) related to 4H leukodystrophy (Table 1).

**TABLE 1:** Whole‐exome sequencing (WES) of the patient

Gene/Transcript (RefSeq)	Variant Location	Variant	Chromosome Position (GRCh37)	Zygosity	Related Phenotypes	OMIM number	Inheritance Pattern
POLR3A NM_007055.3	Intron 13	c.1771‐6C > G	Chr10: 79,769,439	Het	Hypomyelinating leukodystrophy 7 with or without oligodontia and/or hypogonadotropic hypogonadism	607,694	AR
	Exon 31	c.4037G > A p.C1346Y	Chr10: 79,737,372				

*Note*: Compound heterozygote mutation in POLR3A gene related to 4H leukodystrophy was found.

Considering that the radiologic findings and clinical features of the patient were not compatible with the classic 4H leukodystrophy, this diagnosis was not considered at that time. In 2020, reports of mutations in POLR3A gene with movement disorder and striatal involvement were published which matched our patient. Unfortunately, the patient died of respiratory complications earlier and Sanger sequencing could not be performed for confirmation. However, considering the clinical presentation, striatal involvement, and intronic mutation at c.17771‐6C > G which have also been described in two other patients, we believe that this is the accurate diagnosis.

## 3 DISCUSSION

RNA polymerase III (also called Pol III) transcribes DNA to synthesize ribosomal 5 S rRNA, tRNA and other small RNAs. RNA Pol III transcribes the housekeeping genes which are required for all cell types. The regulation of Pol III transcription is primarily linked to the regulation of the cell cycle and cell growth. POLR3A and POLR3B encode the largest subunits of RNA polymerase III including RPc1 and RPc2, respectively.^9^ Mutations in these two genes can lead to a wide range of phenotypes. Previous studies have noted that patients with POLR3A mutation compared to the newly reported POLR3A variant generally demonstrate more severe disorders such as rapid regression and severe neurological defects and shorter life expectancy. However, the disease starts rather later in POLR3A‐mutated patients and most of them achieve independent walking early in life.^10^ Our patient also had a normal history of speech and development before age 2, and the deterioration began gradually afterwards.

The classic phenotype of hypomyelinating leukodystrophy including hypomyelination, hypodontia, and hypogonadotropic hypogonadism (4H syndrome)^10^, ^11^ was not observed in the present patient. Our patient had normal dental and gonadal appearance and function. On the contrary, in the 4H syndrome basal ganglia are spared and ataxia is a chief finding, and dystonia is not a predominant feature^12^ which differ from our patient.

Di Donato et al reported 10 patients with POLR3A mutations, mostly the c.1909 + 22G > A variant, to describe late‐onset spastic ataxia without hypomyelinating leukodystrophy, but they raise other exceptions such as seizures and non‐neurological features, and concluded that further expansions of variants and phenotypic presentations should be investigated.^12^

In recent years, variants of POLR3A mutations without predominant ataxia have been reported which manifest as striatal disorders mostly presenting with dystonia and involvement of putamen, caudate and red nuclei. Harting et al published a retrospective review on clinical, genetic, and MRI findings of nine patients with POLR3A variants and striatal changes, from the patient database at the Center for Childhood White Matter Disorders Amsterdam.^13^ The main clinical feature was extrapyramidal involvement in all the nine patients. One of them had seizures (myoclonic jerks from age 15 months), one had no finding of failure to thrive, and 3 had normal dentition. Main findings on MRI included striatal T2‐hyperintensity/atrophy and involvement of the superior cerebellar peduncles. Interestingly, the authors concluded that the striatal variant is distinct from 4H leukodystrophy and correlates with one of the two intronic variants, c.1771‐6C > G or c.1771‐7C > G, of which our patient had the first one.

Zanette et al reported a 9‐year‐old female patient with severe generalized dystonia, hypotonia, metabolic acidosis, leukocytosis, and dysphagia who had basal ganglia atrophy on brain MRI. Nearly similar to our case, this girl also presented with recurrent pulmonary infections and milestone regression, and was unable to talk at 2 years. Whole‐exome sequencing revealed a compound heterozygous for a missense c.3721G > A (p.Val1241Met) and the splicing region c.1771‐6C > G mutation in POLR3A, which is again very relevant to our patients' findings.^2^

Hiraide et al also reported two sets of compound heterozygous variants in POLR3A, c.1771‐6C > G and c.791C > T, p. (Pro264Leu) and c.1771‐6C > G and c.2671C > T, p. (Arg891*), leading to neuropsychiatric regression and severe intellectual disability in three patients from two families. Both sets shared the c.1771‐6C > G variant, and two of the three patients had dystonia, similar to our patient.^14^

There is also a report of spastic paraplegia and dystonia and minor changes in brain MRI, as a form of adult‐onset POLR3A‐related disorder, in a 35‐year‐old woman.^15^

## 4 CONCLUSION

As described in the literature till now, given that this gene is involved in transcription of many RNA structures, POLR3A mutations can lead to a wide range of phenotypes. Although the most typical known presentation of this mutation is the hypomyelinating leukodystrophy, other phenotypes such as milestone regression, seizure, and dystonia should be taken into consideration as a variant of these genetic mutations.

## AUTHOR CONTRIBUTIONS

**Ali Nikkhah, MD** involved in diagnosing the case and revision of the manuscript. **Sepideh Rezakhani, MD** involved in writing the manuscript.

## CONFLICT OF INTEREST

The authors declare that they have no conflict of interest.

## CONSENT

Written informed consent was obtained from the father of the patient to publish this report in accordance with the journal's patient consent policy.

## Data Availability

Data available on request from outhors
